# Helvétius's challenge: Moral luck, political constitutions, and the economy of esteem

**DOI:** 10.1111/ejop.12471

**Published:** 2019-07-19

**Authors:** Andreas Blank

**Affiliations:** ^1^ Department of Philosophy Alpen‐Adria Universitat Klagenfurt Fakultat fur Kulturwissenschaften Klagenfurt Austria

## Abstract

This article explores a historical challenge for contemporary accounts of the role that the desire of being esteemed can play in exercising social control. According to Geoffrey Brennan and Philip Pettit, the economy of esteem normally has two aspects: it is supportive of virtuous action and it occurs spontaneously. The analysis of esteem presented by the 18th‐century materialist Claude‐Adrien Helvétius challenges the intuition that these two aspects go together unproblematically. This is so because, in Helvétius's view, the desire for esteem is always triggered by sensible interest. In the frequent situations where sensible interest diverges from the demands of virtue, the economy of esteem thus can be spontaneous but will not be supportive of virtue. Helvétius allows for cases of moral luck where sensible interest coincides with the demands of virtue but regards these cases as rare occurrences. This is why he believes that a functioning economy of esteem crucially depends on political constitutions—in which case the economy of esteem can be supportive of virtue but will not be spontaneous.

## 1 INTRODUCTION

During the past two decades, the conception of an economy of esteem has become prominent in political theory. This conception analyzes the quasi‐economic aspects of the role that the desire for esteem and the aversion against contempt has in motivating action: esteem can be understood as a scarce good; its scarcity induces competition; and even if esteem cannot be transferred in any direct way, one can provide a range of esteem services that enhance the esteem in which others are held. In their ground‐breaking work on the economy of esteem, Geoffrey Brennan and Philip Pettit make two claims that sound highly plausible, both regarded in themselves and read in combination with each other: (1) Unless in circumstances of secrecy and oppression, the economy of esteem is supportive of virtuous behavior since if persons act virtuously, “then they get the extra reward of being thought to be virtuous” (Brennan & Pettit, 2004, p. 261). (2) Usually, the economy of esteem originates spontaneously (in the sense that agents need not be controlled from the outside; ibid., p. 262), such that subsequent “institutional design or redesign” can rely on the continued existence of norms that are formed spontaneously (ibid., pp. 285–287).

The first claim allows that, in cases where those who experience the negative consequences of an action know who the relevant agents are, or if they are in a subjugated position, agents will not be motivated by considering whether their action will be esteemed by those affected. At the same time, claim (1) treats such situations as exceptions to the more frequent occurrence of situations where actions are visible and those experiencing their consequences are not dominated. And certainly, one easily can identify a multitude of everyday situations—think of the reasons that people do not free ride in public transportation—where such mechanisms lead to desirable consequences. Brennan and Pettit also point to a wealth of empirical research that documents the occurrence of behavior that creates trust and trustworthiness between agents in the absence of any institutional sanctions (ibid., p. 269). The desire for mutual esteem could explain why these phenomena occur. Claim (2) explains what is so attractive about the economy of esteem as a mechanism of social regulation: If the competition for esteem ordinarily takes place without presupposing any institutional framework, and if the mere anticipation of being regarded unfavorably by others constitutes a powerful motivation for acting, then the economy of esteem can be regarded as an almost cost‐free mechanism of social coordination (Brennan & Pettit, 2000, p. 78).

The idea of an economy of esteem that, more often than not, spontaneously produces virtuous action has not gone without critical responses. One line of criticism, developed by Rae Langton, emphasizes that circumstances of oppression may be more pervasive than Brennan and Pettit acknowledge (Langton, 2009, pp. 273–291). Another line of criticism, developed by Richard H. McAdams, emphasizes how easily the desire to be esteemed motivates individuals to conform to the tastes and preferences of others (McAdams, 2005). As observations concerning what the world is like, both lines of thought may be instructive. But since Brennan and Pettit are aware of the problem that the economy of esteem can work as an “intangible backhand” (Brennan & Pettit, 1993), these lines of criticism seem rather to confirm their central claim that, in the absence of secrecy, domination, and conformity pressure, the economy of esteem works as a beneficial “intangible hand.”

In what follows, I would like to explore a different line of criticism—a line of criticism that does not target claims (1) or (2) in isolation but rather asks whether the *combination* of these claims is as unproblematic as Brennan and Pettit seem to believe. I will take a rather circumstantial, historical route. But this is very much in the spirit of Brennan and Pettit, who emphasize that their view has historical, and especially early modern, predecessors. Perhaps the closest historical analogue to their views can be found in Adam Smith's *Theory of Moral Sentiments* (1759; Smith, 1976, pt. 1, section 2). However, to develop a critical perspective on the combination of claims (1) and (2), looking elsewhere will be more helpful. Here, I will discuss a line of criticism suggested by the treatment of esteem in the thought of the French materialist Claude‐Adrian Helvétius (1715–1771).^1^

Helvétius's central thesis is that esteem always reflects the tastes and interests of the subject who is passing judgment, be it an individual, a group, or a nation. On the basis of this thesis, he offers an analysis of pathologies of esteem that are not restricted to the cases of secrecy and oppression but are rather the outcome of the structure of sensible interest. I will explore these issues in Section 2. Still, Helvétius believes that in exceptional cases, esteem for genuinely moral action is possible. However, he believes that such virtuous actions and the ability to hold them in high esteem spontaneously are the result of a kind of moral luck. In his view, it depends on chance whether or not the natural constitution of an individual causes the tastes and interests of that individual to coincide with the demands of morality. This will be the topic of Section 3. Since the notion of luck implies that lucky events occur rarely, Helvétius argues that the functioning of the desire for esteem as a widespread motivation for action depends on political arrangements connected with esteem and the power to secure sensible pleasures. This will be the topic of Section 4. Section 5 will explore how Helvétius defends his insight into the role of political constitutions for the economy of esteem against the objection that national biases may provide a much more reliable source of self‐esteem. Section 6, finally, will wrap up Helvétius's challenge to the contemporary treatments of the regulative function of the desire for esteem. To put it in a nutshell: In Helvétius's view, the desire for esteem can be a widespread and spontaneous regulative force, but then it will not be supportive of virtue; or it can be spontaneous and supportive of virtue, but then it will not be widespread; or it can be widespread and supportive of virtue, but then it will not be spontaneous.

## 2 ESTEEM AND SENSIBLE INTEREST

In *De l'esprit* (1757), Helvétius addresses two questions that are central to any treatment of esteem: (a) What is the object of esteem? (b) Why do we desire to be esteemed? Helvétius's answers to both questions derive from his account of sensible interest for human motivation. As to the first question, Helvétius claims that “personal interest is the unique and universal ground for appreciating the merit of human actions” (Helvétius, 1795, 1:185). He clarifies that personal interest has several aspects. First, it depends on what we take to be useful for ourselves: “[W]e take more interest in an idea the more this idea is useful for us” (ibid., 1:185). Second, when it comes to ideas that relate to our passions and tastes, we will always prefer those that are flattering to our own passions and tastes over those ideas that challenge them (ibid., 1:186). Helvétius argues that this is so because these ideas “are suitable to justify the high opinion that they all have of the rightness of their mind” (ibid., 1:187)—that is, they are suitable to give us a pleasant feeling about ourselves. Third, he notes that thinking about ideas that diverge from one's own requires effort (ibid., 1:195–196)—that is, they force us into a labor that, for most people, has an unpleasant feeling. Usefulness, vanity, and laziness alike have the consequence that almost everyone esteems in others only ideas that are in conformity with one's own ideas (ibid., 1:195).

Helvétius concedes that we are ready to *express* esteem about such ideas out of respect for public opinion or out of confidence in the opinion of experts. But he takes this to be only “esteem on parole” (*estime sur parole*; ibid., 1:196). By contrast, what he calls “felt esteem” (*estime sentie*) depends on the conformity of ideas just described (ibid., 1:197). In Helvétius's view, the conditions for felt esteem lead to irrational practices of esteem. One aspect could be described as a systematically distorted self‐esteem that results from the fact that our ideas have the highest degree of conformity with our own ideas. The high opinion that we have of ourselves and the admiration that we show for ourselves is not so much the result of pride than of the “necessity in which we are to esteem ourselves in preference over others” (ibid., 1:201). Moreover, since no two humans have exactly similar ideas, we think that we think better than anyone else (ibid., 1:202). This does not exclude the insight that one is much inferior to others in highly specialized skills; still, one believes in one's own superiority over specialists by denying the importance of their skills and by elevating the importance of common sense and social versatility (ibid., 1:204). Another aspect of irrational practices of esteem shows itself in systematically distorted low esteem for others. One example that Helvétius mentions is the contempt between members of different social classes (ibid., 1:227). Another example is the contempt for other historical ages (ibid., 1:324). A further example (to which I will return in Section 5) is the contempt for other nations.

As to the question of why we desire esteem, Helvétius again points to the influence of self‐interest. His thesis is that the passion for esteem is “an effect of physical sensibility,” namely, of the desire for the benefits for our sensible life that being esteemed brings with it (ibid., 2:112). He understands this view as an alternative to the view that we desire the esteem of others because we are uncertain about our personal merits. In particular, he rejects the hypothesis that we seek a sentiment of our own excellence that we cannot get without the feedback from and comparison with others (ibid.). In support, he offers a group of arguments: (a) The first argument starts from a thought experiment: Imagine that there were other inhabited worlds and that some beings with super‐human powers were able to spread your fame in these other worlds. Would you prefer galactic esteem over national esteem? Helvétius suggests that everyone would prefer national esteem (ibid., 2:113). As a related consideration concerning esteem in remote regions of our planet, he suggests this is so because our conationals (in contrast with inhabitants of other worlds and inhabitants of remote regions of our planet) can do something useful for us (ibid., 2:114). (b) Humans show a desire for high political positions even under circumstances where it is politically impossible to achieve much. People have this desire even when they know that they will attract public contempt for their indifference toward public esteem. Hence, in situations where they can procure for themselves personal advantages without gaining esteem, they will prefer these advantages over being esteemed (ibid., 2:113). (c) One is more flattered by esteem from the powerful than from those in low positions; hence, interest in being esteemed is proportional to the advantages that esteem can procure (ibid.). (d) Everyone in France prefers military fame to literary fame because the public gives more esteem to a general than to a philosopher (ibid., 2:111–112). Similarly, almost everyone prefers the esteem of the unenlightened multitude over the esteem of some few enlightened persons because “the larger number of admirers recalls to our mind the enjoyable image of the pleasures that they can procure for us” (ibid., 2:112). (e) Experience with oriental despotism shows that people show contempt for the striving for public esteem when the political system does not offer any rewards for merits but rather discourages actions that document moral and intellectual virtues (ibid., 2:114). From these arguments, Helvétius concludes that the love of esteem reduces to the love of the pleasures arising from being esteemed (ibid., 2:115–116).

In *De l'homme* (published posthumously in 1773), Helvétius returns to these issues and gives them a further edge by claiming that the desire for esteem is grounded in the desire for *power.* He maintains that the interest in happiness is nothing but an interest in having all the means necessary to be happy; and, as he argues, this interest boils down to an interest in power because power is the personal quality sufficient to procure the means necessary for happiness: “The love of power is founded on the love of happiness, and it therefore is the common object of all our desires; also, wealth, honor, fame … respect, justice, virtue … are in us nothing other than love for power disguised under different names” (ibid., 3:337). This is why Helvétius takes the interest in esteem to be nothing but a disguise that the interest in power takes: “The one who spends the night under arms or in the office imagines loving esteem, but that's an error. Esteem is nothing but the name that one gives to the object of one's love, and the thing itself is power” (ibid., 3:340–341).

If this line of argument is coherent, then it would become clear why the malfunctioning of the economy of esteem is inherent in psychological dynamics common to all humans. However, the coherence of Helvétius's argument has recently been questioned by Francesco Toto. As Toto maintains, there is an unresolved tension between Helvétius's view that our desire for esteem is always driven by sensible interest and Helvétius's view that virtuous citizens prefer esteem over wealth and sincere admiration over false flattery (Toto, 2017, pp. 167–194; see Helvétius, 1795, 2:141–143; 2:377–378). Should these descriptions of virtuous citizens be taken to indicate, as Toto suggests, that Helvétius ascribes to their passions “a certain independence from their stipulated physical and utilitarian root” (Toto, 2017, p. 168)? I think that Toto has a viable point when it comes to Helvétius's description of *heroic* virtue. This is so because preferring death to humiliation—a virtue for which Helvétius has high respect (Helvétius, 1795, 2:144; 2:172; 2:185–186)—can hardly be reduced to sensible interest. By contrast, seeking esteem for actions that are in accordance with morality need not be understood from the perspective of heroic virtue. For the very reason that morality reconciles utility and justice, seeking virtue‐based esteem rather than wealth and seeking sincere admiration need not be disconnected from sensible interest. On the contrary, Helvétius maintains that the desire of wealth and the desire of being esteemed are expressions of the *same* kind of desire under different circumstances (ibid., 2:141). Helvétius's analysis of the connection between the desire for esteem and sensible interest thus cannot (perhaps with the exception of his treatment of heroic virtue) be dismissed as incoherent.

Helvétius's point thus seems to be that, whether or not individuals seek esteem, the actual motivation behind their actions is their interest in pleasure and power. This view explains why the economy of esteem can be spontaneous, widespread, but still not conducive to virtue. Such situations occur whenever pleasure in the sameness of ideas arises from shared immoral prejudices and when public esteem procures power to those who act according to shared immoral prejudices. In this way, Helvétius's analysis goes beyond the analysis of the effects of the “intangible backhand” offered by Brennan and Pettit since it offers an explanation of why the malfunctioning of the economy of esteem need not be restricted to situations of oppression and secrecy. Still, as we shall see in the following two sections, Helvétius also allows for the possibility that individuals are spontaneously motivated to fulfill duties of morality, as well as for the possibility that the economy of esteem widely supports virtuous action. The real challenge that he poses for contemporary accounts of the economy of esteem is his insight that the desire for esteem may not support morality spontaneously and in a widespread manner *at the same time.* This is so because he holds a spontaneous disposition toward seeking esteem through virtuous action to be a matter of moral luck. Consequently, he takes a widespread disposition toward seeking esteem through virtuous action to be a matter of political constitutions.

## 3 ESTEEM AND MORAL LUCK

As to the role of luck in our moral lives, Helvétius proposes: “Without despising the vicious man, one must mourn him, and congratulate oneself for a lucky natural constitution …” (ibid., 1:184). Moral luck here is characterized as a kind of natural luck—luck that has brought forth natural dispositions that others are lacking. Among these natural dispositions, Helvétius counts intellectual capacities that are the result of chance in education: “[C]hance has a greater part in our education than we think. It is chance that puts certain objects before our eyes and consequently occasions for us lucky ideas and sometimes leads us to greater discoveries” (ibid., 2:7). Accordingly, he draws attention to the fact that no one receives exactly the same education because circumstances never coincide in two persons (ibid., 2:10). Helvétius never gives any definition of the concept of chance. But he observes that a vast amount of small impressions goes into education in early age, most of which are entirely unintended by the educators (ibid., 3:30). Some of them are the unintended side effects of intentional actions, such as when a child pays attention to the shades of color of pots on the window board while being locked up in a room as a form of punishment (ibid., 3:31–32). It is the effects that such forgotten small impressions have on conscious ideas that Helvétius describes as the effects of chance:

But what influence can a difference in instruction occasioned by some light difference in surrounding objects have on minds? Well, does one ignore that a small number of different ideas that are combined with ideas that two humans already have in common, can produce a difference in their entire way of seeing and judging? (Ibid., 3:30)

From a contemporary perspective, Helvétius's conception of moral luck could be described as a conception of “constitutive” luck—that is, of luck that explains what an individual is like. Helvétius's remarks about the differences in education also make it clear that the comparison that he has in mind is nothing as sophisticated (and problematic) as a comparison between an individual in the actual world and individuals in nearby possible worlds,^2^ but rather the comparison between different individuals in the actual world. The morally lucky are those who, through their education, have received a combination of impressions that occurs rarely. And these impressions are not only outside the intentional control of those who develop virtuous character traits but partly also outside the intentional control of those who educate them.

Still, describing Helvétius's conception of moral luck in this way seems to make it vulnerable against a recent criticism of the concept of constitutive luck. As David Enoch and Andrei Marmor argue, if character traits cannot be regarded as the object of intentional control, then individuals could not be thought worthy of praise or blame for such character traits (Enoch & Marmor, 2007, pp. 429–430). One possible response to this problem has been suggested by Judith Andre, who points out that Aristotelian ethics offers the possibility to evaluate character traits morally even in situations where agents did not contribute anything to developing these traits. This is so because Aristotelian virtues are excellences of character that are naturally good in the life of humans, and Aristotelian vices are natural defects of character, whether or not these traits have been formed voluntarily (Andre, 1983, p. 205). Of course, the specifically Aristotelian framework is not helpful for reading Helvétius. But Andre's point can be generalized to other, non‐Aristotelian versions of virtue ethics, as well. This is particularly relevant for Helvétius, who develops a theory of virtue within the context of his adoption of a version of natural law theory.

Helvétius is not usually considered to be one of the early modern natural law thinkers; however, as Sophie Audidière and Ann Thomson have emphasized, in a postscript to *De l'esprit*, Helvétius describes the project of this work to be a version of natural law theory (Audidière, 2004, p. 352; Thomson, 2016, pp. 252–254).^3^ On natural rights of peoples (*droits naturels des nations*), Helvétius there remarks: “These rights oppose themselves to the despotism of a power that is entirely foreign to primordial convention and to the fundamental laws of the constitution of societies” (Helvétius, 1795, 5:280). What he does reject is a metaphysical idea of natural law as something that is innate or derives from the divine will and intellect: “Experience only proves too much to which point artificial minds have deceived humans through the false interpretation of the abstract idea of natural law” (ibid., 5:280–281). By contrast, Helvétius holds that reason is what makes it possible to apprehend natural law (ibid., 5:281). In his view, sensible interest is necessary for motivating us to act according to what reason demands:

It is not only the light of reason, nor only the inner sentiment of the law alone that guides humans in their deliberations. Inner sentiment makes them attentive; reason enlightens them; but in addition, it is necessary that sensations represent the objects that provide the motivations of physical goods or evils, of moral goods or evils, which determine the decisive acts of the will. (Ibid., 5:282)

This commitment to a non‐theological version of natural law theory is highly relevant for Helvétius's views on the goals of philosophy and politics. As he suggests, the task of the philosophers is to think about the morality relating to legislation, and the task of the legislator is to find out what, under ever changing circumstances, is useful for a given community (ibid., 5:337). “But the one and the other grounds the institution of public laws on human nature and on the law of laws, and on the physical dynamics of human actions, and on justice that is co‐essential for the general well‐being of society” (ibid., 5:337–338). The philosopher and the legislator who fulfil this task thus exemplify the character traits that give substance to the idea of constitutive moral luck. Because these character traits are genuinely useful both for the community and for those who have these traits, they are a legitimate object of positive moral evaluation, even if they are not the outcome of intentional control.

The morally lucky therefore can develop mutual esteem that is in accordance with the demands of morality. Helvétius describes our readiness to judge the actions and ideas of others according to the norms of morality as the outcome of a multiplicity of accidental factors:

Interest presents objects to us only from the side that it is useful for us to apperceive them. When one judges according to public interest, it is not so much due to the justness of one's mind or the justice of one's character that one should render honor than to the chance that has placed one in circumstances where we have an interest to see as the public sees. (Ibid., 1:226–227, note 1)

When actions are judged according to public interest, Helvétius holds that those who pass such judgments fulfill nothing other than “the passion that an enlightened pride gives them for virtue and, consequently, nothing other than obeying, like every other society, the law of personal interest” (ibid., 1:207). Even in such cases, “interest is the only distributor of esteem” (ibid., 1:410). More specifically, “he justice of our judgments and our actions is never anything but the lucky coincidence of our interest with the public interest” (ibid., 1:226). Consequently, Helvétius holds that “every author who gives new ideas to the public can hope for the esteem of only two kinds of humans: either young people who, because they have not yet adopted any options, still have the desire and the leisure to instruct themselves; or those whose mind, being a friend of truth and analogous to the mind of the author, already suspects the existence of the ideas that he presents to him” (ibid., 1:201).

For Helvétius, esteeming ideas that one recognizes as being superior to one's own thus is a genuine possibility.^4^ However, it is a possibility that only can be realized when we encounter others who search for the truth in a way similar to how we search for the truth and who entertain ideas that have enough connection with the ideas that we had before and that enabled us to anticipate their ideas, even if not in a fully articulated form. In this way, even esteeming ideas that one recognizes as superior to one's own can function as a pleasant confirmation of inclinations that we can be proud of. “Noble and enlightened pride” allows persons of the latter kind “to be attached to their beliefs without being opinionated” and “to preserve in their mind the suspending of judgment, which there leaves free access to new truths” (ibid., 1:187). As Helvétius explains, “humans of this kind always esteem in others ideas that are true, enlightened, and suitable to satisfy the passion that an enlightened pride gives them for the true” (ibid.). For this reason, he takes it to be important to cultivate the right kind of pride: “Pride is the seed of so many virtues and talents that one must not hope to destroy it, nor even to try to weaken it, but only to direct it toward honorable things” (ibid., 1:226). In this way, esteem between the morally lucky is motivated by a kind of sensible interest—the interest in triggering a pleasant self‐related emotion. Even the morally lucky thus are not motivated by anything like an intrinsic value of virtue or of esteem.

## 4 ESTEEM AND POLITICAL CONSTITUTIONS

Moral luck, for Helvétius, thus explains why practices of mutual esteem that are both spontaneous and supportive of virtue can occur. At the same time, it explains why such practices occur only in exceptional cases. In this sense, his account of moral luck does not explain much about our ordinary practices of esteem. This is exactly what makes his considerations concerning moral luck so interesting from the perspective of the contemporary debate concerning the economy of esteem: While Brennan and Pettit believe that a morally beneficial economy of esteem occurs both widely and spontaneously, Helvétius's considerations concerning moral luck raise the suspicion that cases of practices of mutual esteem that are both beneficial and spontaneous may not be widespread.

What is more, Helvétius's conception of moral luck explains the presence of a claim that has been noted by his interpreters but that remains puzzling if read independently of the issue of moral luck. The claim that I have in mind is Helvétius's thesis that the “administration of the monies of honors” is mainly a *political* task (ibid., 2:198).^5^ If one assumes, as Toto does, that Helvétius ascribes to virtuous citizens (even to those who are not heroes) a desire for esteem that is independent of a physical or a utilitarian basis, it would seem inexplicable why Helvétius would put so much weight on political planning. In this context, Toto diagnoses a further unresolved tension in Helvétius's thought: According to Toto, Helvétius oscillates between the ideal that political regulation could bind the economy of esteem to the pursuit of public interest and the empirical observation that in despotic regimes, the economy of esteem leads to the result that the most powerful receive the most esteem, no matter how virtuous they are (Toto, 2017, p. 189; see Helvétius, 1795, 1:256; 2:98–99).

These interpretive puzzles dissolve once one takes the issues of sensible interest and moral luck into account. The very concept of luck implies that, due to the nature of chance, most individuals do not fall into the category of the morally lucky. The crucial consequence that Helvétius draws from the rare occurrence of moral luck and the frequent distortion of our judgment through sensible interest is that it is the task of legislators to force subjects to observe the law of nature (ibid., 5:298). Helvétius regards this as a realistic project because morality is “bound to our well‐understood interest” (ibid., 5:297). As a remedy for destruction of societies, he recommends “to interest individuals, through good laws, to concur to the general well‐being of the society in which they live” (ibid., 5:298). Helvétius claims that his conception of moral motivation does not exclude freedom. As he argues, even the insight into the rewards and punishments that the legislator has attached to laws and their transgression forms part of rational deliberation that leads to choosing the course of action that is most strongly in the personal interest (ibid., 5:318). But clearly, even if there may be a sense in which actions guided by rewards and punishments can be regarded as free, they cease to be spontaneous.

At the same time, the virtue‐supportive function of the economy of esteem, in Helvétius's view, is not just result of *politics* but the result of specific political *constitutions.* It is here that the connection between the desire for esteem and sensible interest becomes crucial. Helvétius acknowledges that the desire for esteem can motivate the search for truth; but he notes that the love for the true is always subordinate to the love of happiness. In his view, it is impossible to love in the true anything other than the means for increasing happiness. Hence, few will search for truth under a government where truth is despised (ibid., 3:322). He also offers an argument for why the economy of esteem in a despotic regime cannot get off the ground:

[I]f honors derive their price from the way in which they are administered, and if in the Orient sultans are the distributors of honor, one senses that they must often discredit honors through the bad choice of those whom they decorate with them. Also, in those countries, honors … cannot vividly flatter pride because they are rarely united with glory, which is not at the disposal of princes but of peoples; for glory is nothing other than the expression of public recognition. (Ibid., 2:196)

Generally, Helvétius holds that under a government that debases talents, ingenious persons are entirely the result of chance or of the memory that one has preserved of the honor that has been bestowed on virtue and talent in the past (ibid., 3:287). By contrast, under a government that honors merit, love for fame is not the result of chance but of the constitution of the state (ibid., 3:287–288). Therefore, he takes it to be a political task to trigger the love for public esteem (ibid., 3:291). But it is a task that can be accomplished only in particular political constitutions: “Why is fame regarded as a plant of republican soil, which, degenerated in despotic countries, never grows there with a certain strength? It is because in fame one loves properly only power, and in an arbitrary government, all power disappears in the face of the power of the despot” (ibid., 3:314). On the contrary, “[i]n a free nation, public reputation and esteem is a power, and the desire for this esteem there becomes consequently a powerful principle of action” (ibid., 3:328). But even in a republican constitution, the economy of esteem always is a fragile interplay between political agency and public opinion. This is why those who are active in defining positions of honor have to develop a fine discernment for when a certain kind of honor has become too common since “honors owe their price only to the opinion of humans” (ibid., 2:198). Since this is such a delicate task, Helvétius proposes the creation of institutions that have the task of regulating the distribution of honors in a society (ibid.). Under a republican constitution, the desire for esteem thus in fact plays a central role in social regulation; if the political institutions define positions of honor in such a way that personal and public interests are united, the desire for esteem also may be largely supportive of virtuous action; but the fact that esteem leads to enough power to secure sensible interest may be shaped more profoundly by political institutions than we may be aware of.

## 5 ESTEEM AND NATIONAL BIASES

How plausible, from an empirical point of view, is Helvétius's view that the economy of esteem is, to a large degree, an outcome of political institutions? This question is pressing in the light of extensive psychological research that documents the extent to which human judgments succumb to a variety of biases. Why should we think that the esteem in which persons hold themselves and others could be more strongly influenced by a structure of institutionalized rewards and sanctions than by prejudices that are favorable for their self‐esteem? Let me focus here on the example of national biases, which play a significant role both in contemporary psychological research and in Helvétius's moral philosophy.

Of course, even giving an overview over the literature on the psychology of nationalism would go beyond the scope of the present paper. Luckily, however, there are two excellent overview articles that cover the literature from the mid‐1950s to the early 2000s (Brock & Atkinson, 2008; Spinner‐Halev & Theiss‐Morse, 2003), which can be complemented by some newer studies. Some empirical findings seem to be particularly telling with a view to Helvétius. Most studies on the psychology of nationalism use a conceptual distinction between (1) national identification that includes feelings of national superiority and derogation of other nations and (2) national identification that involves a positive evaluation of national institutions and values. This distinction has led to surprising results concerning the influence of national identification on subjective well‐being: Compared with the absence of national identification, identification of type (1) is correlated with *lower* levels of subjective well‐being (although feelings of pride compensate for this effect to some degree), while identification of type (2) is correlated with *higher* levels of subjective well‐being (Reeskens & Wright, 2011). More specifically, contrary to what philosophical nationalists have claimed, identification of type (1) has *not* shown any widespread increase in self‐esteem (although some individuals show the opposite tendency; Blank, 2003). What is more, identification of type (1) is correlated with authoritarian attitudes (ibid.)—that is, the readiness to accept the decisions of superiors without critical debate (which is presumably contrary to the interests of those who entertain such attitudes). Identification of type (1) is also correlated with high levels of economic inequality within a nation—which supports the diversion theory of nationalism according to which nationalistic sentiments are triggered to divert the attention of the economically disadvantaged from their real situation (Solt, 2011; which is, again, presumably contrary to their interests). By contrast, the finding that identifications of type (2) enhance subjective well‐being corresponds to what group psychology has documented on a large scale: Usually, membership in a positively evaluated group enhances the self‐esteem of group members but, usually, this effect is entirely independent of feelings of superiority or out‐group derogation (Brewer, 2001).

Helvétius, too, distinguishes between two kinds of national identification, one that involves unjustified feelings of national superiority and one that involves an appreciation of political institutions that unite personal and public interests. As to the first kind of national identification, Helvétius is clear about the pervasive occurrence of unjustified national biases. One of his explanations for the occurrence of national biases invokes the sensible interest that we have in esteeming only ideas that are similar to our own ideas. As he argues, this psychological mechanism explains why one esteems or disesteems the ideas of other societies according to their correspondence with or divergence from one's own ideas (Helvétius, 1795, 1:219). In his view, the resulting attitudes toward other nations are systematically flawed:

This folly, common to all nations, … leads them not only to disdain the ways of living and the customs different from their own, but also leads them to regard the superiority that some have over others as a gift of nature—a superiority that they owe only to the political constitution of their state. (Ibid., 1:369)

Accordingly, he holds that “contempt for a nation is always an unjust contempt” (ibid., 1:370–371). But no matter how unjust contempt between nations may be, he nevertheless describes it as being grounded in human nature (ibid., 1:226). That the objects of our esteem and the motivation for seeking esteem are determined by sensible interest, he notes, can also explain why personal interests deriving from laziness and fear can lead us to esteem cruelties in one's own nation that one despises in other nations. For instance, the coexistence of widespread contempt for the ritual practices of Maya priests with widespread esteem for the cruel methods of the Inquisition is a vivid illustration for how much interest can distort esteem for other nations (ibid., 4:271). In addition to the interests deriving from laziness and fear, Helvétius also identifies the interest of vanity to explain national biases: “Each nation will always exalt the virtues that it owes to the form of its government to a gift of nature. The interest of vanity advises it do so: and who could resist the advice of interest?” (ibid., 1:373).

However, conceptualizing the persistence of national biases in the framework of sensible interests also opens up a plausible conception of how such biases could be overcome. What is needed are the workings of *other* sensible interests. As Helvétius holds, this is exactly what can be achieved through identification with a political constitution that unites personal and public interests. He regards this kind of national identification itself as a result of political constitutions. For instance, he claims that the “spirit of patriotism” found in England is due to the form of government (in addition to geographical position that prevented an invasion from abroad during the Interregnum; ibid., 1:372). If a political constitution unites personal and public interests, among other things through the rewards connected with merit, it is easy to see how sensible interests are served by identifying with this constitution. This is why Helvétius holds that republican constitutions lead to civic spirit (*l'esprit de citoyen*), while monarchic constitutions lead to an indifference toward public affairs (ibid., 1:371–372).

Helvétius's view that national identification connected with an appreciation of one's own political institutions could serve the interest in self‐esteem better than entertaining national biases is supported by the psychology of nationalism. The empirical findings mentioned above indicate that national identification based on feelings of superiority and derogation of other nations actually serves very poorly the interests of those who take this attitude. This is why it is not unrealistic for a political constitution that uses the desire for esteem to connect what is personally useful with what is publicly useful will create a mentality that does not try to derive self‐esteem from national bias but rather from actions that contribute to the well‐being of the community and that is perceived and rewarded as such. And, as we have seen, there is empirical support for the assumption that national identification based on a positive evaluation of national institutions and values in fact increases subjective well‐being. All that Helvétius would add to this empirical finding is the reminder that such a form of patriotism presupposes political constitutions that really unite private and public interests.

## 6 CONCLUSION: HELVÉTIUS'S CHALLENGE

What is interesting about Helvétius's considerations concerning the desire for esteem is that they envisage a possibility that, as far as I can see, has not been discussed in the contemporary debate about the economy of esteem: the possibility that, if the desire for esteem supports virtuous action *spontaneously*, this is mainly a matter of moral luck. The contemporary discourse about moral luck is almost completely separated from the discourse about the economy esteem. The only exception to this is a brief but thought‐provoking article by Jeffrey Fry. As Fry points out, there are two kinds of situations where significant others can have a lucky influence on self‐esteem: personal interactions in early childhood that give growth to self‐esteem and personal encounters that help to recover self‐esteem independently of being successful in competitions. Fry argues that such situations could be described as involving “grace” rather than “chance”: while chance implies the absence of intentional action, interactions of the type described are intentional and the proper object of thankfulness (Fry, 1996). As we have seen, Helvétius acknowledges the possibility of seeking esteem for naturally good actions, and he certainly believes that such actions deserve public thankfulness (*reconnaissance*; *Ouevres*, 2:191). But he presses the analysis of such actions one step further by asking about the conditions on which they depend, and some of these conditions—physiology and the long history of personal experiences—inevitably involve factors that are not intended outcomes of actions. This is why Helvétius sees a close connection between the virtue‐supportive function of the economy of esteem and moral luck. In his view, persons with lucky dispositions can develop esteem for virtuous actions, as well as the desire to be esteemed for virtuous actions, just because this is what turns out to be pleasurable for them.

Due to the role of chance, such situations will occur rarely. In the absence of political steering, Helvétius therefore argues that personal interests will lead most individuals to systematically flawed practices of esteem—ranging from inflated self‐esteem, to derogation of others, to contempt for other nations. This is why he holds that political arrangements are crucial for the proper working of the economy of esteem: the distribution of esteem must be regulated in such a way that esteem remains a scarce good; it must be regulated by criteria of justice that are sensitive to the usefulness and difficulty of certain activities; and the rewards for esteem must be regulated in such a way that esteem gives enough power to fulfill sensible interests. Helvétius also offers an analysis of what happens to the economy of esteem in situations of oppression. What he has in mind is not the observation that the members of the oppressing group will not be concerned about the esteem of the oppressed. Rather, he notes that despotic regimes annihilate the economy of esteem even within the oppressing group. As he argues, this is so because under despotic rule, esteem is no guarantee for power and, hence, no guarantee for sensible pleasure. People know this and, except for the morally lucky, will not be motivated by a desire for esteem.

An observation that Brennan and Pettit adduce to support their spontaneity thesis in fact offers further empirical support for Helvétius's contrary thesis. As Brennan and Pettit observe, the failures of free markets in post‐communist countries show that fair market practices cannot occur without a tradition of civic values (Brennan & Pettit, 2004, p. 269). Yet does this consideration support the claim that esteem for civic virtue is largely a spontaneous phenomenon where such esteem occurs? I think that Helvétius gives us good reasons to think that the contrary is the case. The absence of esteem for civic virtues seems to be a consequence of decades of dictatorial rule—hence, of political constitutions. Conversely, if esteem for civic virtues was a largely spontaneous phenomenon, one would expect it to occur widely once political obstacles have been removed—contrary to what can be observed. This observation lends plausibility to the view that what is lacking in post‐communist economies is a comprehensive set of political institutions and educational practices that connects the desire for esteem with sensible interest. And after all, Brennan and Pettit emphasize that the empirical studies that they cite in favor of the occurrence of behavior that creates trust and trustworthiness among agents specifically concern traditions of *civic* behavior—hence, behavior that takes place in the context of specific political constitutions (ibid.).

Of course, Brennan and Pettit accept the view that political institutions could shape the economy of esteem: In their view, “centralized initiatives” and “perhaps even formal laws” could be used “to promote suitable kinds of publicity for potentially estimable or dis‐estimable behavior, to establish modes of presentation that will activate the required form of esteem or disesteem, and to ensure that people are predisposed to be moved by corresponding, esteem‐related motives” (ibid., 287). The divergence between Helvétius and Brennan and Pettit thus does not concern the feasibility of institutional influences on the economy of esteem. Rather, it concerns the question of priority: While for Brennan and Pettit, the existence of widespread, reliable, and virtue‐supporting practices of mutual esteem and disesteem is the basis upon which subsequent institutional design and redesign depends, Helvétius holds that the existence of widespread, reliable, and virtue‐supporting practices of mutual esteem and disesteem depends upon institutional design.

Helvétius's challenge could thus be wrapped up as follows: (a) As long as our personal interests are not in line with the duties of morality, our practices of esteem will be systematically flawed. Such practices may be spontaneous and widespread, but they do not contribute to virtuous action. (b) An economy of esteem that is spontaneous and supportive of virtue is a genuine possibility, but due to its dependence on moral and intellectual luck, it will be a rare occurrence. (c) An economy of esteem that is widespread and supportive of virtue is a genuine possibility, but moral luck will have to be supplemented by political planning and education that guarantees that virtuous action is rewarded with an amount of power that is sufficient for fulfilling sensible interest. In such cases, the economy of esteem will be spontaneous only to a small extent. If there is something to these three considerations, then the connection between the claim that the economy of esteem is usually supportive of virtue and the claim that it is mainly a spontaneous phenomenon may be more problematic than is obvious on first sight. This is a challenge that still deserves a response.

## ENDNOTES

1 All references to Helvétius's writings will be to Helvétius (1795). I have checked the text against Helvétius (2006) and Helvétius (2011). All translations from Helvétius are my own; however, I have also consulted with Helvétius (1777). On publication history, see Warner Smith (1997); for an overview of Helvétius's philosophy, see Longué (2008); and on Helvétius's political thought, see Spitz (2000, pp. 53–78); Wooton (2000).

2 On the problems of a counterfactual analysis of constitutive luck, see Latus (2003, pp. 470–472).

3 This line of interpretation offers an alternative to the more strongly conventionalist interpretation of Helvétius's theory of law developed in Punzi (2003, pp. 244–246).

4 This is overlooked by Audidière, who takes Rousseau's objection that it is possible “to feel inside me that this man has more spirit than me, and to accord him an esteem felt in my heart as superior to that which I have for myself” to be a definitive refutation of what Rousseau “felt as the diminution of the human and individual subject in Helvétius's philosophy” (Audidière, 2016, p. 999; for Rousseau, see Masson, 1911, p. 108; translation from Audidière, 2016, p. 997).

5 As emphasized by Audidière, 2006, p. 157, and Toto, 2017, pp. 180–185.
